# Genome-Wide microRNA Profiling of Rat Hippocampus after Status Epilepticus Induced by Amygdala Stimulation Identifies Modulators of Neuronal Apoptosis

**DOI:** 10.1371/journal.pone.0078375

**Published:** 2013-10-25

**Authors:** Zhen Sun, Jin-Tai Yu, Teng Jiang, Meng-Meng Li, Lin Tan, Qun Zhang, Lan Tan

**Affiliations:** 1 Department of Neurology, Qingdao Municipal Hospital, Taishan Medical University, Qingdao, China; 2 Department of Neurology, Qingdao Municipal Hospital, School of Medicine, Qingdao University, Qingdao, China; 3 Department of Neurology, Qingdao Municipal Hospital, Nanjing Medical University, Qingdao, China; University of Florida, United States of America

## Abstract

MicroRNAs (miRNAs) are small and endogenously expressed non-coding RNAs that negatively regulate the expression of protein-coding genes at the translational level. Emerging evidence suggests that miRNAs play critical roles in central nervous system under physiological and pathological conditions. However, their expression and functions in status epilepticus (SE) have not been well characterized thus far. Here, by using high-throughput sequencing, we characterized miRNA expression profile in rat hippocampus at 24 hours following SE induced by amygdala stimulation. After confirmation by qRT-PCR, six miRNAs were found to be differentially expressed in brain after SE. Subsequent Kyoto Encyclopedia of Genes and Genomes pathway analysis indicated that most of the predicted target genes for these six miRNAs were related to neuronal apoptosis. We then investigated the dynamic changes of these six miRNAs at different time-point (4 hours, 24 hours, 1 week and 3 weeks) after SE. Meanwhile, neuronal survival and apoptosis in the hippocampus after SE were evaluated by Nissl staining and terminal deoxynucleotidyl transferase-mediated dUTP end-labeling assay. We found that the expression of miR-874-3p, miR-20a-5p, miR-345-3p, miR-365-5p, and miR-764-3p were significantly increased from 24 hours to 1 week, whereas miR-99b-3p level was markedly decreased from 24 hours to 3 weeks after SE. Further analysis revealed that the levels of miR-365-5p and miR-99b-3p were significantly correlated with neuronal apoptosis after SE. Taken together, our data suggest that miRNAs are important modulators of SE-induced neuronal apoptosis. These findings also open new avenues for future studies aimed at developing strategies against neuronal apoptosis after SE.

## Introduction

Epilepsy is a common neurological disorder affecting almost 0.5% to 1% of the population. The pathogenesis of epilepsy is associated with selective neuronal losses and mossy fiber sprouting, which results in the structural and functional alterations of hippocampus and limbic system [1], [2]. However, the underlying molecular mechanisms of epilepsy still remain unclear. Meanwhile, despite of the recent advances in development of antiepileptic drugs, the treatment of epilepsy still remains as a major challenge [3].

MicroRNAs (miRNAs) belong to a family of non-coding small RNAs that modulates gene expression at the translational level [4]. In mammalians, approximately 50% of all miRNAs are expressed in the brain [5], [6] and many of them may play critical roles in multiple neurologic diseases including epilepsy [7]. However, their precise functions in epilepsy remain largely unclear. Status epilepticus (SE) is a clinical emergency, which is defined as continuous unremitting seizure lasting for more than 30 minutes [8]. Several studies have characterized the expression profiles of miRNAs in mouse model of SE induced by lithium, pilocarpine or kainic acid [9]–[16], and identified that many miRNAs were involved in the pathogenesis of epilepsy in direct or indirect ways. It should be noted that chemoconvulsants were employed to induce SE in these studies, which may have additional effects on miRNA expression, thus affecting the accuracy of the results.

It is generally accepted that the amygdala stimulation model is the most suitable animal model to study SE, which could well mimic behavioral, electroencephalographic, and morphological phenomena of SE in humans [8], [17]. In the current study, by using high-throughput sequencing, we investigated the miRNA expression profiles in rat hippocampus after SE induced by amygdala stimulation. In addition, the association between differentially expressed miRNAs and neuronal apoptosis was also evaluated in this study.

## Materials and Methods

### Animals

Adult male Wistar rats (260–300 g, provided by the Experimental Animal Center, Qingdao University, China) were used in this study. They were housed in a standard animal room with a 12 hours light/dark cycle and given free access to food and water.

### Ethics Statement

This study was carried out in strict accordance with the recommendations in the Guide for the Care and Use of Laboratory Animals of the National Institutes of Health. All procedures were approved by the Animal Care and Management Committee of Qingdao University (permit No. QUEC-130205). Remarkably, efforts were made to minimize the number of animals used in the study and their sufferings.

### Electrode implantation and SE induction

Rats were anesthetized for surgery with 10% chloral hydrate (3.5 mL/kg, i.p.). Once anesthetized, rats were positioned in a stereotaxic apparatus (Stoelting, USA). Electrodes were implanted into the right hemisphere aimed at the basolateral amygdale as described previously (AP: −3.0 mm; L: −4.8 mm; V: −8.8 mm) [18], [19]. The above coordinates were determined according to the atlas of Paxinos and Watson (1986). The electrodes were made of stainless steel wires (0.16 mm in diameter, A.M. Systems, USA) insulated with teflon-coating except for 0.5 mm at the tip. The tip separation was 0.7–0.8 mm. The electrodes were connected to a miniature receptacle, which were fixed to the skull using dental cement anchored with stainless steel screws. All control animals underwent identical surgeries. After electrodes implantation, the animals were treated with penicillin for 5 days to prevent infection and allowed to recover for 10 days.

After a post-operative period of 10 days, SE was induced by stimulating the lateral nucleus of the right amygdala for 20 min. The stimulation consisted of a 100-ms train of 1-ms biphasic square wave pulses (400 µA from peak to peak) that were delivered at 60 Hz every 0.5 sec using a ML1101 electronic stimulator. Electroencephalograms (EEGs) of the right amygdala were recorded with a digital amplifier (AD Instrument, Bio Amp, USA). After 20 min of continuous stimulation, the stimulation was interrupted, and the behavioral and electrographic activity of animals was observed for 60 sec. If the behavior of the animals indicated the presence of epileptic activity (head nodding or limb clonus), observation was continued for another 5 min. If an animal did not meet the criteria of clonic SE (continuous EEG epileptiform spiking and recurrent clonic seizures), stimulation was resumed and the behavior of the animal was checked again after 5 min. Once the criteria of SE were achieved, no further stimulation was given. Stimulation period never exceeded 40 min. Control rats were handled in the same manner while did not receive any electrical stimulation.

### Brain tissue preparation

Rats were anesthetized with 10% chloral hydrate (3.5 mL/kg, i.p.) at 4 hours, 24 hours, 1 week and 3 weeks after the induction of SE (n = 6 per group). They were transcardially perfused with ice-cold saline to remove intravascular blood components, which avoided potential contamination of blood miRNA in tissue samples. The brains were rapidly removed and hemisected along the midline. The left hippocampi were frozen in liquid nitrogen for RNA isolation while the right hippocampi were divided into consecutive frozen sections (20 µm thick).

Total RNA isolation was carried out using the mirVana™ miRNA Isolation Kit (Ambion, Austin, TX, US) following the manufacturer's instructions. The quality and quantity of RNA are assessed on the Agilent Bioanalyzer for presence of 5, 5.8, 18S bands.

### Construction of small RNAs library and deep sequencing

Total hippocampal RNA from experimental rats at 24 hours after SE as well as control rats was pooled separately and used for miRNA sequencing. This time point was selected based on previous findings about miRNA expression profile following SE [9], [12]. Next, the small RNAs were ligated to a 5′ and a 3′ adaptor sequentially and then converted to cDNA by reverse transcription. The two generated small cDNA libraries were amplified by PCR with primers complementary to the adaptor sequences. Finally, the libraries were deep sequenced directly using the Illumina Cluster Station and Genome Analyze (Illumina Inc, CA, USA) at Beijing Genomics Institute (BGI) according to the manufacturer's protocol [20]. The deep sequencing data have been deposited in NCBI SRA database and are accessible through GEO series accession number GSE50940 (http://www.ncbi.nlm.nih.gov/geo/query/acc.cgi?acc=GSE50940). The differences in the quantities of miRNAs were determined by comparing the log2-ratio of the experimental rats (24 hours post-SE) and control rats copies.

### Confirmation and quantification of miRNAs by qRT-PCR Analysis

To validate the initial results of deep sequencing and to perform further investigation in this study, we selected miRNAs that met the following criteria for additional qRT-PCR analysis: (a) having at least 30 copies in either experimental or control group (24 hours post-SE). (b) Showing fold-change (log_2_) >2 or fold-change (log_2_) <-2 between these two groups (*P*<0.01 and *P*≠0). As a result, five up-regulated miRNAs (miR-874-3P, miR-20a-5p, miR-345-3p, miR-365-5p and miR-764-3p) and one down-regulated miRNA (miR-99b-3p) were screened out for subsequent qRT-PCR confirmation. Each reaction mixture of reverse transcription consists of 1 µL purified total RNA, 10 µL of 2×miRNA Reaction Buffer Mix, 2 µL of miRNA PrimeScript RT Enzyme Mix, 2 µL of 0.1% BSA, and RNase-free H_2_O up to a total volume of 20 µL. The 20 µL volume reactions were then incubated for 60 min at 37°C and for 5 sec at 85°C. qPCR was carried out using a CFX-96 Realtime System (BIO-RAD) in a 20 µL reaction volume. All reactions were done in triplicate. The 20 µL PCR reaction contained the following: 1 µL of miRNA RT product, 10 µL SYBR remix Ex Taq™, 2 µL of miRNA forward primer, 2 µL of miRNA reverse primer, 0.5 µL reference dye, and 4.5 µL ddH_2_O. The reaction parameters were incubation at 95°C for 30 sec, then at 95°C for 5 sec, and 60°C for 31 sec. The median quantitation cycle (Cq) is defined as the cycle number at which the fluorescence signal exceeded a pre-determined threshold. A relative fold change in expression of the target gene transcript was determined using the comparative cycle threshold method (2^−ΔΔCq^). The expression of the U6 snoRNA gene was used as internal control. In addition, qRT-PCR was also used to investigate the dynamic alterations of the six selected miRNAs expressions at 4 hours, 24 hours, 1week and 3weeks after SE. Experiments were performed in triplicate.

### Bioinformatics analysis

Potential target genes of these six miRNAs were predicted using the miRNA target prediction databases including RNAhybrid and miRanda [21]–[23]. In addition, the Kyoto Encyclopedia of Genes and Genomes (KEGG) pathway database was used to filter the enriched pathways of miRNA targets.

*Nissl staining and terminal deoxynucleotidyl transferase-mediated dUTP end-labeling (TUNEL) assay*

Nissl staining was employed to detect surviving neurons. For Nissl staining, the frozen slices were stained with 0.5% cresyl violet at 50°C for 5 min. Only the neurons with violet nucleus and the intact morphology were counted as surviving neurons. Besides, the TUNEL assay was used to detect neuronal apoptosis. TUNEL assay was performed by a commercial kit according to the instructions provided by the manufacturer (Roche Co., Germany). TUNEL-positive neurons with condensate nuclear were identified as apoptotic neurons. Cell counting was performed on five randomly selected non-overlapping fields in CA1 and CA3 region of hippocampus per slide. The survival index was defined as follows: surviving index (%)  = 100× (number of surviving neurons/ total number of neurons). Furthermore, the apoptosis index was defined as follows: apoptosis index (%)  = 100× (apoptotic neurons/total neurons).

### Statistical analysis

Statistical analysis was performed using the SPSS 13.0 software. Differences between groups were assessed by independent-samples t-test or one-way ANOVA analysis followed by LSD post hoc test. The correlation between the miRNA levels and apoptosis index was analyzed using Pearson's correlation analysis. All data are expressed as mean±SD. *P*<0.05 was considered statistically significant.

## Results

**Figure 1:**
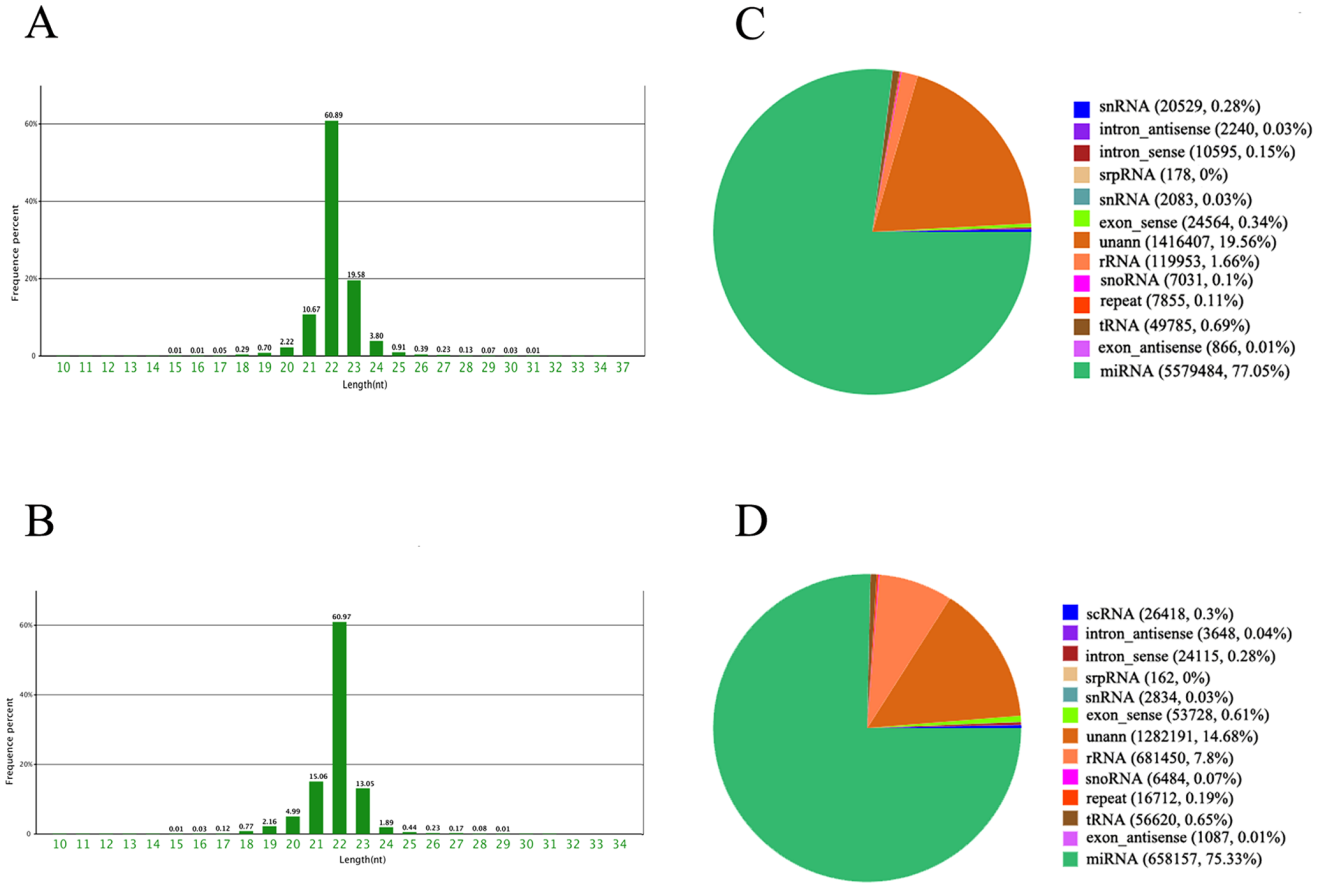
Distribution of the sequencing reads and classification of small RNAs. (**A**) Distribution of the non-redundant sequencing reads from the experimental group (at 24 hours after SE). (**B**) Distribution of the non-redundant sequencing reads from control group. (**C**) Classification of the sequenced small RNA tags from the experimental group (at 24 hours after SE). (**D**) Classification of the sequenced small RNA tags from control group.

In total, 7,321,408 and 8,839,947 raw reads were obtained from the experimental group (24 hours after SE) and control group, respectively. As demonstrated by Figure 1A and 1B, the most abundant size class was 22 nt, which accounted for 60.89% (experimental group) and 60.97% (control group) of the total reads, respectively. After discarding low-quality sequences, sequences shorter than 18 nucleotides, and single-read sequences, 7,241,570 and 8,736,906 clean reads of 18–30 nt from the experimental and control groups were selected for further analysis. Among the clean reads, 5824104 sequences from the experimental group and 7086380 sequences from the control group mapped perfectly to the rat genome, accounting for 77.05% (experimental group) and 75.33% (control group) of the total reads, respectively. Meanwhile, 5579484 reads (experimental group) and 6581457 reads (control group) were found to be similar to known miRNAs (Figure 1C and Figure 1D). The rest of the sequences were found to be other types of RNA, including non-coding RNA, tRNA, rRNA, snRNA or snoRNA.

**Figure 2:**
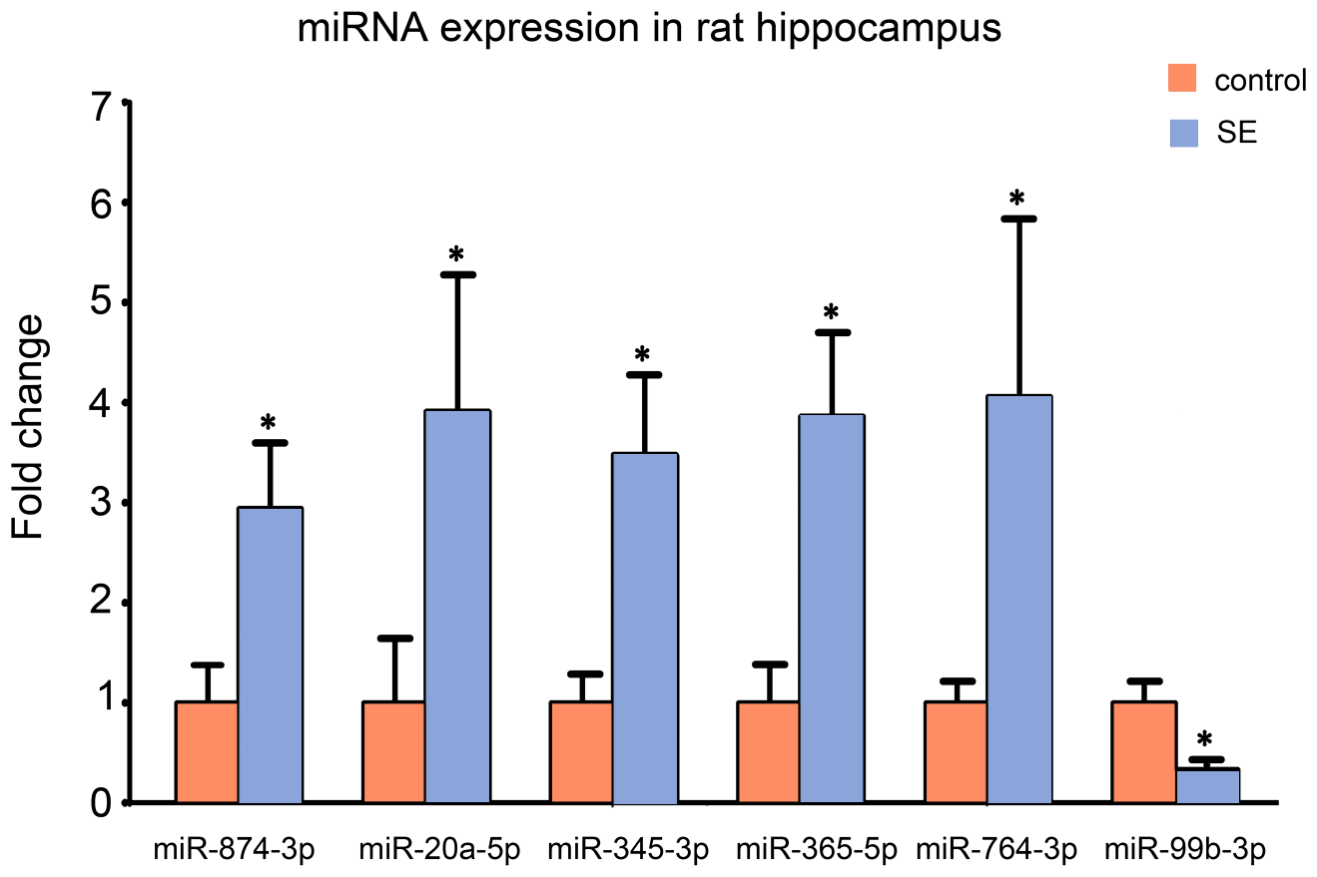
Validation of six differentially expressed miRNAs using qRT-PCR at 24 hours after SE. The levels of six differentially expressed miRNAs (miR-874-3p, miR-20a-5p, miR-345-3p, miR-365-5p, miR-764-3p and miR-99b-3p) in the rat hippocampus at 24 hours after SE were confirmed using qRT-PCR. The expression of all genes was normalized to the levels of U6 snoRNA. Data are presented as the mean ± S.D.; *P<0.05 versus control group; n = 6 per group

After analyzing the initial results of deep sequencing, six deferentially expressed miRNA (miR-874-3p, miR-20a-5p, miR-345-3p, miR-365-5p, miR-764-3p and miR-99b-3p) were screened out based on the criteria mentioned in Materials and Methods, and qRT-PCR was performed to confirm their alterations after SE. As shown in Figure 2, the expression of miR-874-3p, miR-20a-5p, miR-345-3p, miR-365-5p, miR-764-3p was significantly increased after SE (*P*<0.05), whereas the levels of miR-99b-3p was markedly decreased (*P*<0.05). These findings were in agreement with results from miRNA sequencing.

**Figure 3:**
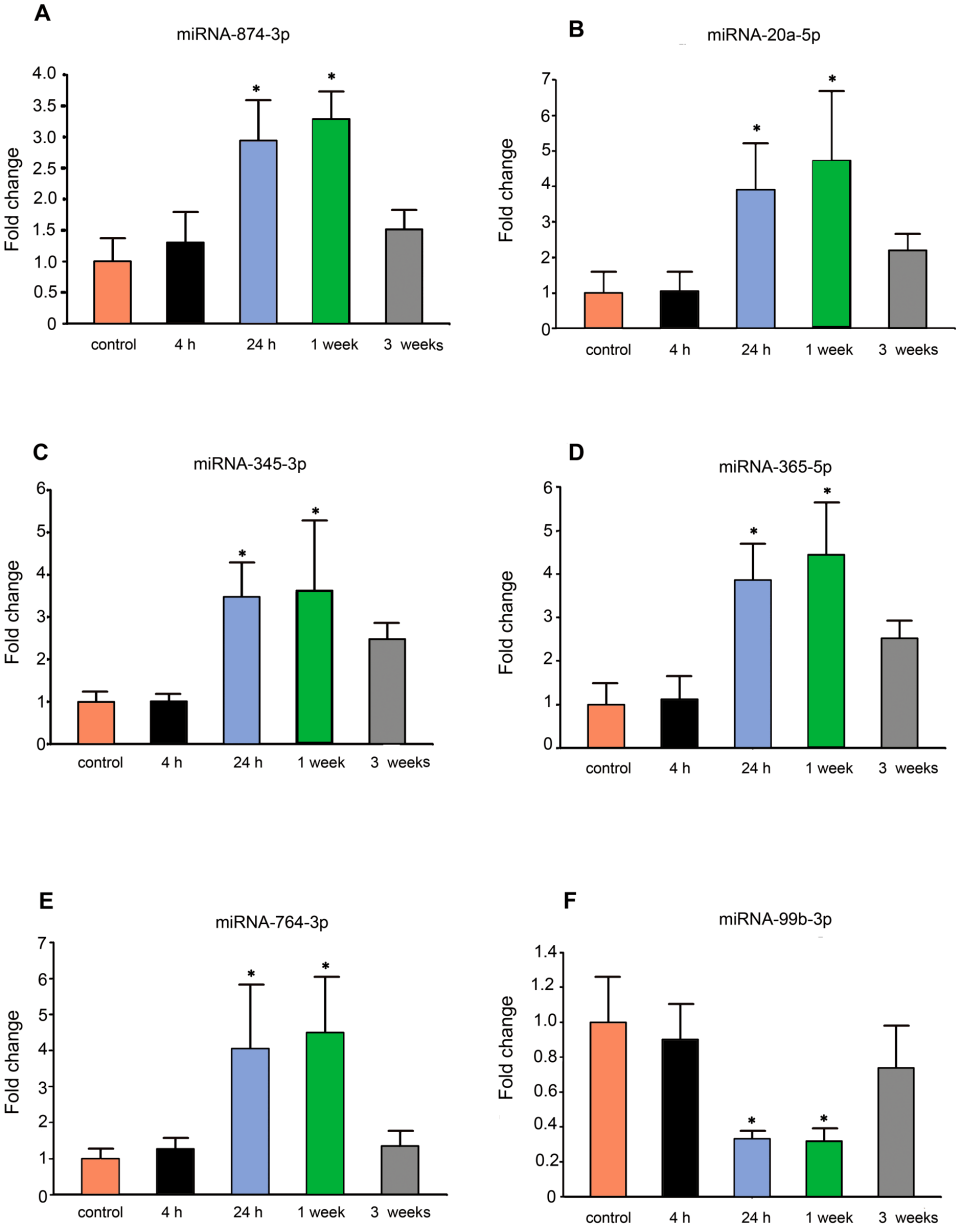
The dynamic alterations of six differentially expressed miRNAs at different time-point after SE. (**A**) Measurement of miR-874-3p expression in rat hippocampus at 4 hours, 24 hours, 1 week and 3 weeks after SE. (**B**) Measurement of miR-20a-5p expression in rat hippocampus at 4 hours, 24 hours, 1 week and 3 weeks after SE. (**C**) Measurement of miR-345-3p expression in rat hippocampus at 4 hours, 24 hours, 1 week and 3 weeks after SE. (**D**) Measurement of miR-365-5p expression in rat hippocampus at 4 hours, 24 hours, 1 week and 3 weeks after SE. (**E**) Measurement of miR-764-3p expression in rat hippocampus at 4 hours, 24 hours, 1 week and 3 weeks after SE. (**F**) Measurement of miR-99b-3p expression in rat hippocampus at 4 hours, 24 hours, 1 week and 3 weeks after SE. The expression of all genes was normalized to the levels of U6 snoRNA. Data are presented as the mean ± S.D.; *P<0.05 versus control group; n = 6 per group.

To investigate the dynamic changes of miRNA expression after SE induced by amygdala stimulation, we detected the expression of six miRNAs using qRT-PCR at four time points (4 hours, 24 hours, 1 week and 3 weeks) after SE induced by amygdala stimulation. The results showed that the expression of these six miRNAs changed in a time-dependent manner. The expression of miR-874-3p, miR-20a-5p, miR-345-3p, miR-365-5p and miR-764-3p were significantly increased at 24 hours after SE (*P*<0.05), peaked at 1 week (*P*<0.05) and returned to the basal levels at 3 weeks after SE (Figure 3A-3E). Furthermore, the expression of miR-99b-3p started to decrease at 24 hours after SE (*P*<0.05), reached its bottom at 1 week (*P*<0.05) and returned to basal levels at 3 weeks after SE (Figure 3F).

**Figure 4:**
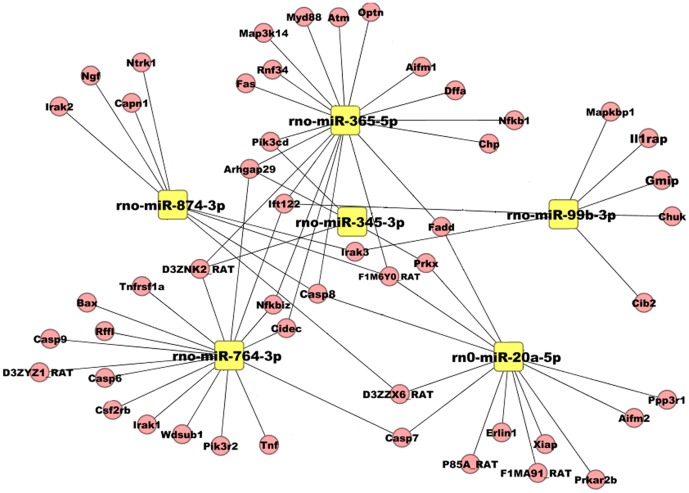
Genes potentially targeted by six differentially expressed miRNAs. Boxes in yellow represent miRNAs, whereas cycles in red represent mRNAs.

To explore the biologic function of the six miRNAs that screened out by our analysis, we first used the RNAhybrid and miRanda algorithm to obtain a list of potential target genes for these six miRNAs. Subsequent KEGG pathway analysis found that most of the predicted target genes were associated with neuronal apoptosis, including Casp, Irak, and Fadd [24]–[26] (Figure 4). This observation suggested that these six miRNAs may modulate neuronal apoptosis after SE.

**Figure 5:**
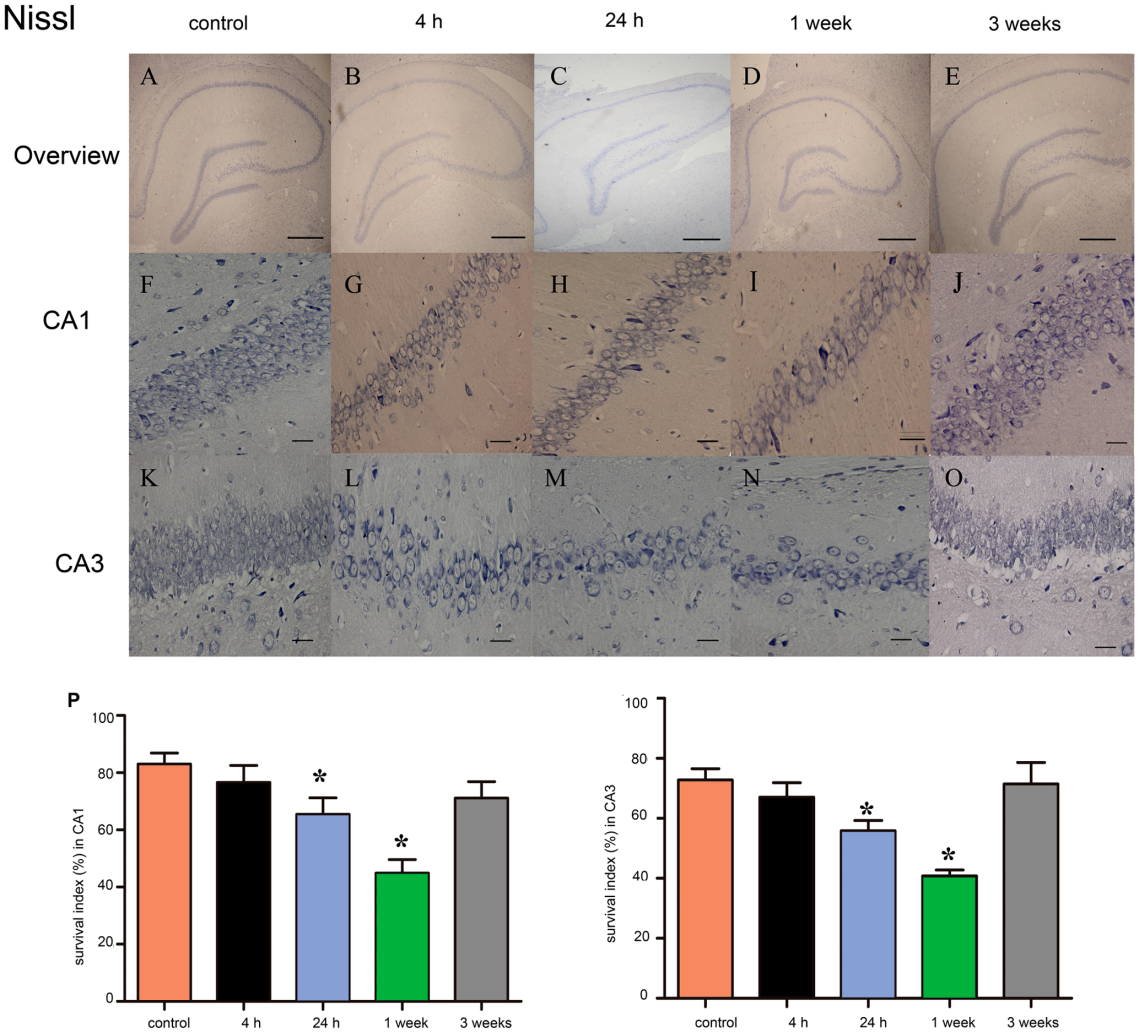
Identification of neuronal survival by Nissl-staining in rat hippocampus at different time-point after SE. (**A–E**) Representative photo of Nissl-staining in rat hippocampus at 4 hours, 24 hours, 1 week and 3 weeks after SE. Scale bars: 500 µm. (**F–J**) Representative photo of Nissl-staining in the CA1 region of rat hippocampus at 4 hours, 24 hours, 1 week and 3 weeks after SE. Scale bars: 50 µm. (**H–O**) Representative photo of Nissl-staining in the CA3 region of rat hippocampus at 4 hours, 24 hours, 1 week and 3 weeks after SE. (**P**) Survival index in the CA1 region at different time-point after SE. (**Q**) Survival index (%) in the CA3 region at different time-point after SE. Data are presented as the mean ± S.D.; * *P*<0.05 versus control group; n = 6 per group.

**Figure 6:**
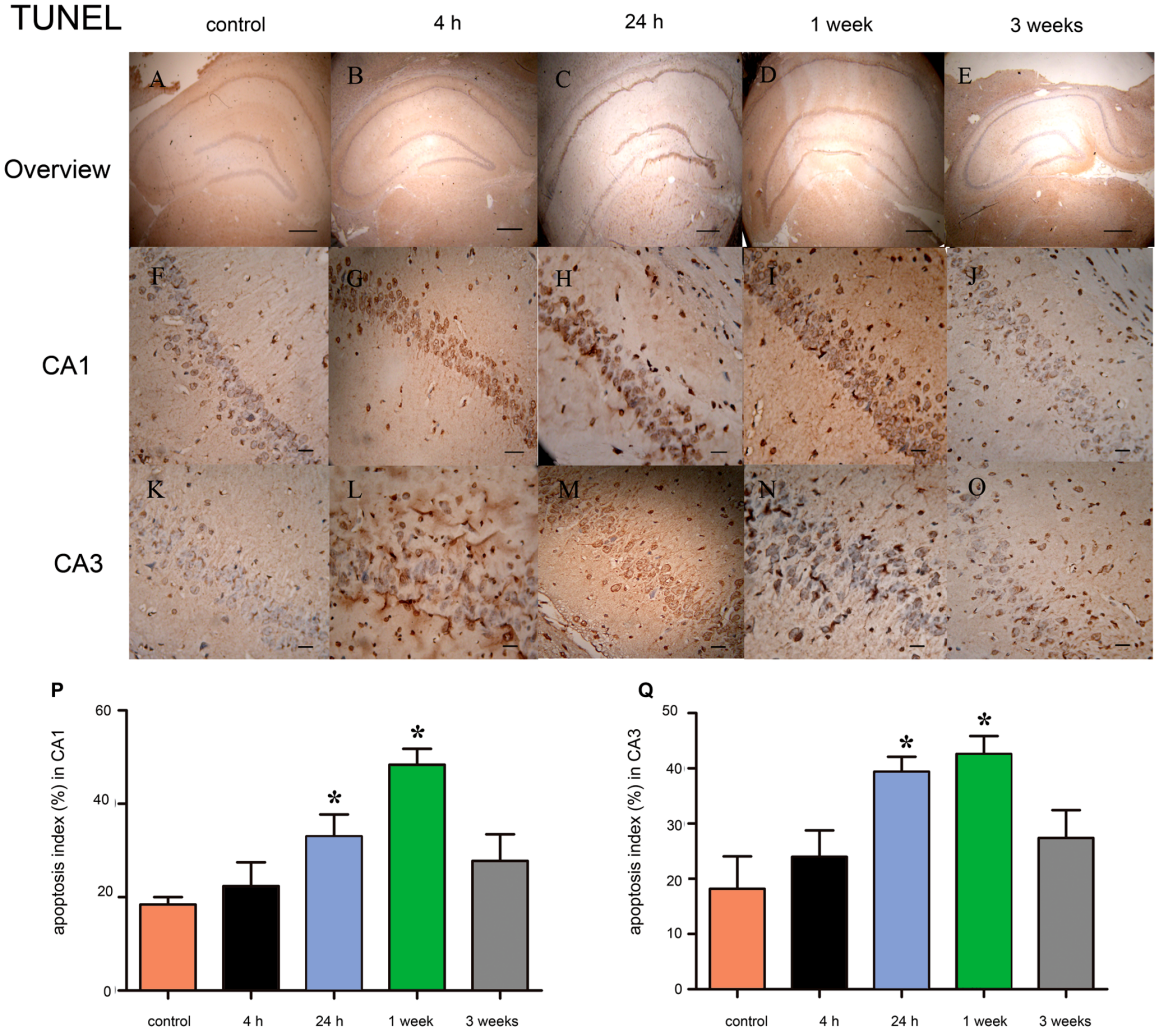
Identification of neuronal apoptosis by the TUNEL-assay in rat hippocampus at different time-point after SE. (**A–E**) Representative photo of TUNEL-staining in rat hippocampus at 4 hours, 24 hours, 1 week and 3 weeks after SE. Scale bars: 500 µm. (**F–J**) Representative photo of TUNEL-staining in the CA1 region of rat hippocampus at 4 hours, 24 hours, 1 week and 3 weeks after SE. Scale bars: 50 µm. (**H–O**) Representative photo of TUNEL-staining in the CA3 region of rat hippocampus at 4 hours, 24 hours, 1 week and 3 weeks after SE. Scale bars: 50 µm. (**P**) Apoptosis index (%) at different time-point after SE in the CA1 region. (**Q**) Apoptosis index (%) in the CA3 region at different time-point after SE. Data are presented as the mean ± S.D.; * *P*<0.05 versus control group; n = 6 per group.

We then assessed survival index and apoptosis index using Nissl staining and TUNEL assay in the CA1 and CA3 regions of the rat hippocampus at 4 hours, 24 hours, 1 week and 3 weeks after SE. Nissl staining showed that neurons in the control group displayed integrative and well-maintained morphology (Figure 5F and 5K). At 4 hours after SE, a moderated but non-significant decrease in the survival index was observed in CA1 and CA3 regions of experimental group (Figure 5G, 5L and 5P). The reduction in survival index in the CA1 and CA3 regions became significant at 24 hours after SE (Figure 5H, 5M and 5P; *P*<0.05), reached its bottom at 1 week (Figure 5I, 5N and 5P; *P*<0.05), and returned to the baseline at 3 weeks after SE (Figure 5J, 5O and 5P; *P*>0.05). In order to investigate whether apoptosis contributed to neuronal loss after SE, we performed TUNEL assay to evaluated apoptosis index in the CA1 and CA3 regions of the rat hippocampus. At 4 hour after SE, a small but non-significant increase in apoptosis index was observed in CA1 and CA3 regions of experimental group (Figure 6G and 6L). The apoptosis index was significantly increased at 24 hours after SE (Figure 5H and 5M, *P*<0.05), peaked at 1 week (Figure 6I and 6N; *P*<0.05), and returned to the basal levels value at 3 weeks after SE (Figure 6J and 6O; *P*>0.05). These results indicated that apoptosis represents part of the mechanisms contribute to the neuronal loss after SE.

**Figure 7:**
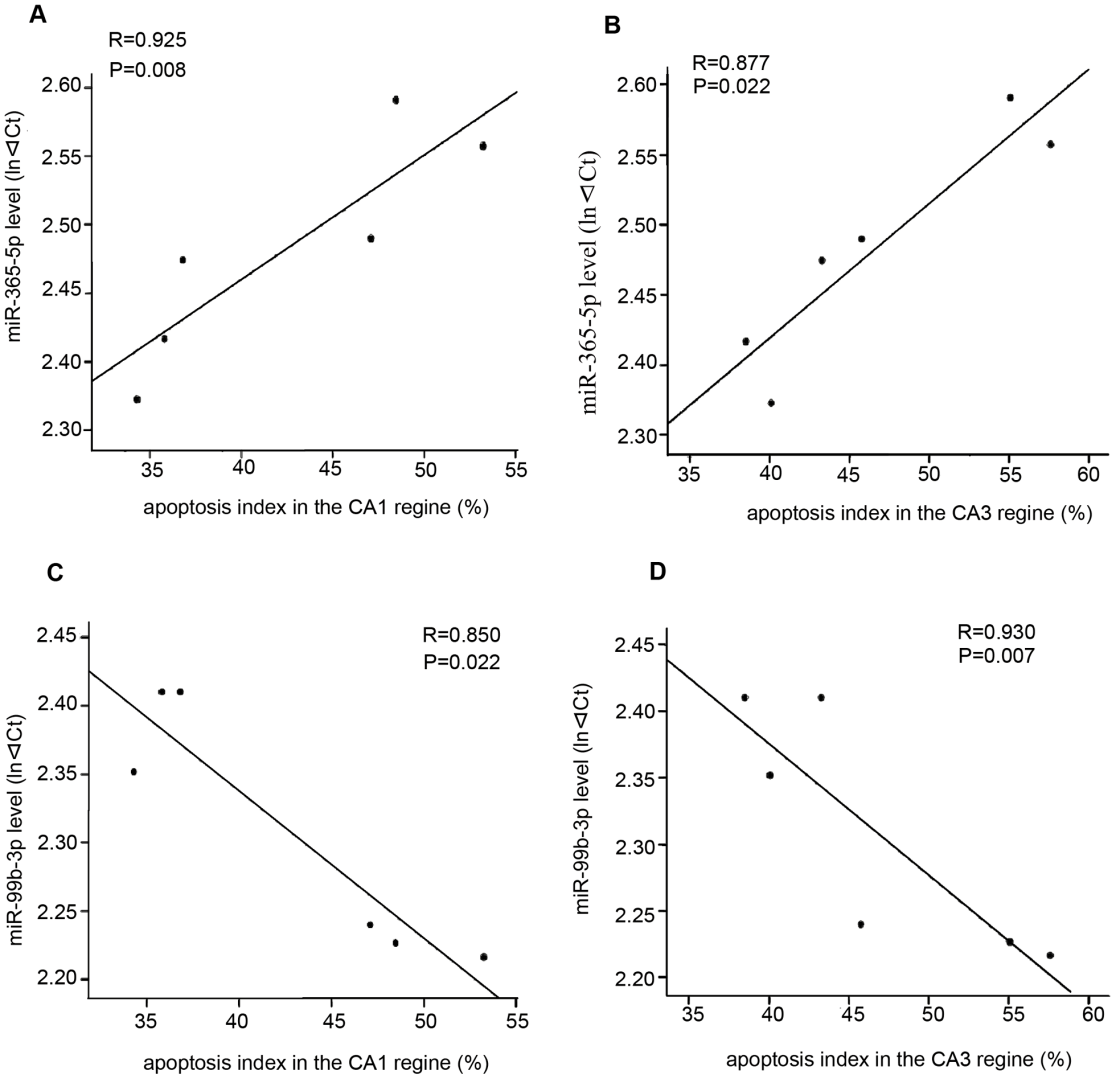
Correlations between levels of miR-365-5p and miR-99b-3p and apoptosis index. (**A,B**) The correlation between miRNA-365-5p and apoptosis index in the CA1 and CA3 regions after SE. (**C, D**) The correlation between miRNA-99b-3p and apoptosis index in the CA1 and CA3 regions after SE.

To further evaluate whether the selected miRNAs were involved in neuronal apoptosis after SE, we investigate the correlation between these six miRNAs and the apoptosis index in the rat hippocampus at 1 week after SE. Pearson's correlation analysis suggested a positive correlation between miR-365-5p and apoptosis index (Figure7A and 7B, CA1: *R* = 0.925, *P* = 0.008; CA3: *R* = 0.877, *P* = 0.0218). We also observed a negative correlation between miR-99b-3p and apoptosis index (Figure7C and 7D, CA1: *R* = 0.850, *P* = 0.032; CA3: *R* = 0.930, *P* = 0.007). Taken together, these data confirmed that miR-345-3p and miR-99b-3p was involved in the pathogenesis of SE by modulating neuronal apoptosis.

## Discussion

In the current study, we provided the first evidence that SE induced by amygdala stimulation led to profound changes in the miRNA expression profile in hippocampus, as six miRNAs were found to be differentially expressed at 24 hours after SE. The results from our study were partly consistent with a previous study by Risbud et al., which characterized expression profile of miRNAs in rat model of SE induced by pilocarpine [11]. For instance, the expression of miR-20a was found to be up-regulated whilst the miR-99b level was significant reduced in both of these two studies. However, the expression of miR-874-3p, miR-345-3p, miR-365-5p, and miR-764-3p were found to be increased in our study, whereas the levels of these four miRNAs stayed unchanged in study from Risbud et al. These differences might attribute to different SE models, experimental methods as well as the criteria for miRNA screening in these two studies. Nevertheless, these findings indicated a possible role of miRNA in the pathogenesis of SE.

Interestingly, the subsequent KEGG pathway analysis indicated that these six miRNAs were targeted to apoptosis-related genes. To confirm the role of these six miRNAs in neuronal apoptosis, we investigate the dynamic change of them in rat hippocampus at different time point after SE. As expected, the alteration pattern of these miRNAs were well matched with that of neuronal apoptosis from 4 hours to 3 weeks following SE. More importantly, we found that the expression of miR-365-5p and miR-99b-3p were significantly correlated with neuronal apoptosis after SE. As a form of programmed cell death, apoptosis was found to directly contribute to neuron losses and brain injury after SE [27]-[29]. SE-induced apoptosis can be triggered by activation of intrinsic pathway components including pro-apoptotic proteins belonging to Bcl-2 and caspase family. In addition, the extrinsic pathway is also found to be engaged following experimental SE and triggers neuronal apoptosis [30], [31]. In the current study, KEGG analysis indicated that the genes potentially targeted by these six miRNAs were involved in either intrinsic or extrinsic pathway of apoptosis, providing further evidence that these miRNAs could regulate neuronal apoptosis after SE. These findings were in agreement with previous studies, which demonstrated expression of several miRNAs was dramatically changed after SE and provided modulation on neuronal apoptosis. In previous studies, miR-34a and miR-132 were found to be increase after SE, and silencing miR-34a or miR-132 by microinjection of antagomirs led to the inhibition of caspase-3 expression, which was accompanied by reduction in apoptotic neurons, indicating a positive regulation of these two miRNA on neuronal apoptosis [12], [13]. Interestingly, in a recent study from McKiernan et.al, silence of miR-184, another miRNA that significantly up-regulated after SE, led to an increase in the counts of apoptotic neurons, suggesting a negative regulation of miRNA on neuronal apoptosis [14].

In summary, we characterized miRNA expression profile in rat hippocampus at 24 hours after SE induced by amygdala stimulation and screened out six differentially expressed miRNAs, which were associated with signaling pathway of neuronal apoptosis. More importantly, miR-365-5p and miR-99b-3p have been revealed to be significantly correlated with neuronal apoptosis. These findings indicated that miRNAs may contribute to the pathogenesis of SE by providing modulation on neuronal apoptosis, and more studies should be performed to further investigate the underlying mechanisms by which these miRNAs regulates neuronal apoptosis.
